# Efficacy of labral repair, biceps tenodesis, and diagnostic arthroscopy for SLAP Lesions of the shoulder: a randomised controlled trial

**DOI:** 10.1186/1471-2474-11-228

**Published:** 2010-10-07

**Authors:** Øystein Skare, Cecilie Piene Schrøder, Olav Reikerås, Petter Mowinckel, Jens Ivar Brox

**Affiliations:** 1 Lovisenberg Deakonal Hospital, Lovisenberggaten 17, 0440 Oslo, Norway; 2 Orthopaedic Department, Oslo University Hospital-Rikshospitalet, Sognsvannsveien, 0027 Oslo, Norway, and University of Oslo; 3 Pediatric Department, Oslo University Hospital-Ullevål, 0407 Oslo, Norway

## Abstract

**Background:**

Surgery for type II SLAP (superior labral anterior posterior) lesions of the shoulder is a promising but unproven treatment. The procedures include labral repair or biceps tenodesis. Retrospective cohort studies have suggested that the benefits of tenodesis include pain relief and improved function, and higher patient satisfaction, which was reported in a prospective non-randomised study. There have been no completed randomised controlled trials of surgery for type II SLAP lesions. The aims of this participant and observer blinded randomised placebo-controlled trial are to compare the short-term (6 months) and long-term (2 years) efficacy of labral repair, biceps tenodesis, and placebo (diagnostic arthroscopy) for alleviating pain and improving function for type II SLAP lesions.

**Methods/Design:**

A double-blind randomised controlled trial are performed using 120 patients, aged 18 to 60 years, with a history for type II SLAP lesions and clinical signs suggesting type II SLAP lesion, which were documented by MR arthrography and arthroscopy. Exclusion criteria include patients who have previously undergone operations for SLAP lesions or recurrent shoulder dislocations, and ruptures of the rotator cuff or biceps tendon. Outcomes will be assessed at baseline, three, six, 12, and 24 months. Primary outcome measures will be the clinical Rowe Score (1988-version) and the Western Ontario Instability Index (WOSI) at six and 24 months. Secondary outcome measures will include the Shoulder Instability Questionnaire (SIQ), the generic EuroQol (EQ-5 D and EQ-VAS), return to work and previous sports activity, complications, and the number of reoperations.

**Discussion:**

The results of this trial will be of international importance and the results will be translatable into clinical practice.

**Trial Registration:**

[ClinicalTrials.gov NCT00586742]

## Background

The glenoid labrum contributes to stability by increasing joint concavity and dept of the glenohumeral joint socket. The superior glenoid labrum of the shoulder joint is a common site of injury and degeneration[1,2]. Because it is related to the intraarticular insertion of the long head of the biceps tendon, injuries are common in throwing athletes. These lesions are often associated with other shoulder injuries such as rotator cuff tears, glenohumeral instability or impingement, but they also may be due to an isolated injury. Snyder et al. used the term SLAP (superior labrum anterior posterior) to describe these lesions, and they classified the lesions into four categories [1]. Type II SLAP lesions, which occur most frequently, are characterised by the combined detachment of the superior labrum and biceps tendon from the peripheral edge of the glenoid. Surgical treatment includes reattachment of the labrum with the use of staples, metal screws, bioabsorbable tacks, and bioabsorbable anchors. Alternatively, tenodesis of the biceps tendon is performed, by inserting the tendon in the bicipital groove of the humeral head, either with suture anchors or interference screws.

Systematic reviews have analysed the value of diagnostic tests for SLAP-lesions [3,4]. Recently, a systematic review summarised the current evidence about the outcome of type II SLAP repair [5]. Twelve studies, including 10 to 50 patients each, with at least 2-years of follow-up, were included; two studies compared two different surgical methods, two studies were prospective, while ten were retrospective cohort studies. There were no randomised trials. The percentage of patients classified as good to excellent varied from 40 to 94%. A return to their previous level of sports activity varied from 20 to 94%. Despite these unpredictable results and a lack of evidence from properly designed studies, shoulder surgeons worldwide perform type II SLAP repairs.

The aforementioned systematic review recommended that future studies should be prospective in nature and they should at least use a longitudinal prospective cohort design. Because uncontrolled studies have the potential to provide a distorted view of treatment results, and non-randomised trials are liable to produce biased results, we designed a prospective, randomised, double-blind, sham-controlled trial.

### Aims

There are two aims of this randomised placebo-controlled trial:

1) Compare the short-term (6 months) efficacy of labral repair, biceps tenodesis, and placebo (diagnostic arthroscopy), for alleviating pain and improving function for type II SLAP lesions.

2) Compare the long-term (2 years) efficacy including the number of reoperations.

## Methods/Design

### Trial design

**Figure 1:**
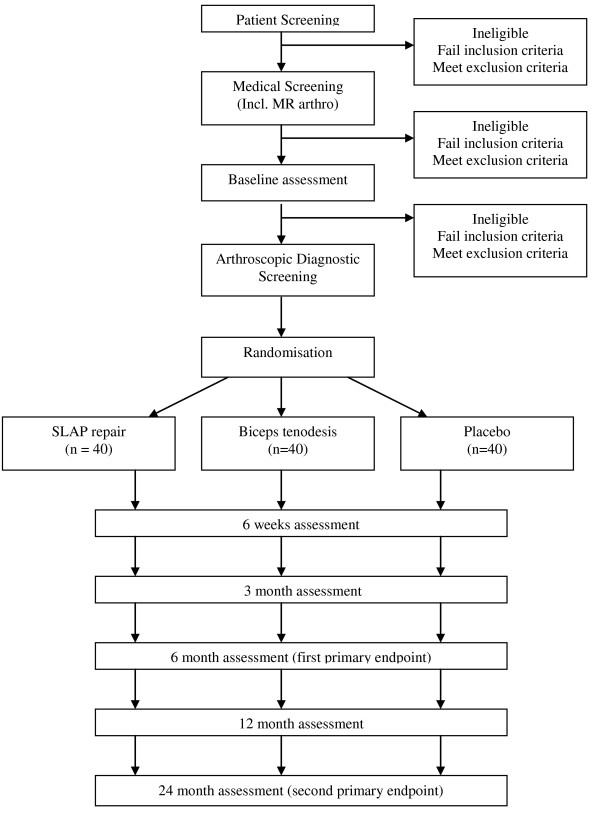
**Diagram of Recruitment and Participation Process**. Placebo is sham surgery (diagnostic arthroscopy). All groups had standard postoperative rehabilitation.

This is a participant and observer blinded randomised placebo-controlled trial with a 2-year follow-up (Figure 1).

### Ethics

Ethics approval for this study has been received from the Ethics Committee Health Region Southeast, Oslo, Norway.

### Participants

Participants will be recruited from general practitioners, physiotherapists, manual therapists, and from departments of orthopaedic surgery or physical medicine and rehabilitation. To increase the awareness of the trial, health care providers will be invited to attend lectures on shoulder complaints with a focus on the current study.

All potential participants will be screened to determine their eligibility according to the following inclusion and exclusion criteria. For inclusion, participants should be aged 18 to 60 years and have a history of type II SLAP lesions or clinical signs suggesting the presence of a type II SLAP lesion, and an MR arthrography that documents the type II SLAP lesion [6]. Finally, the diagnosis should be verified at arthroscopy. One experienced shoulder surgeon and one experienced manual therapist will perform clinical examinations of the patients. Patients should have at least one positive sign of a SLAP lesion (positive O'Brien test [7], positive Crank test [8], or painful apprehension test [9]).

A thorough clinical examination will be performed to exclude possible candidates with differential diagnoses. The clinical examination will include tests for impingement [10], pain or weakness on isometric tests of abduction and external rotation [11,12], tests for apprehension and relocation [13], scapular dyskinesis [14], and arthritis of the acromioclavicular joint [15]. Possible candidates will have an MR arthrography evaluated by a radiologist experienced in shoulder imaging. In addition, conventional x-rays including outlet view will be conducted to exclude patients with major acromioclavicular or acromial spurs.

Exclusion criteria include previous surgery for SLAP lesions, SLAP lesions with concomitant labral cysts [16], previous surgery for recurrent shoulder dislocation or SLAP lesions, clinical and radiological signs of arthritis of the acromioclavicular [15] or the glenohumeral joints, ruptures of the rotator cuff or biceps tendon [11], synovial chondromatosis, fibromyalgia, major somatic or psychiatric disease, and patients that are not able to understand Norwegian or unwilling to accept one of the treatment alternatives.

### Randomisation

Participants who fulfil the inclusion criteria, and consent to take part in the trial after they have received the oral and written information, will be randomised to receive labral repair, biceps tenodesis, or placebo (diagnostic arthroscopy) treatment. An independent statistician will use the method of permuted blocks for random allocation after the final inclusion criteria are met. Treatment allocation will be organised by an independent secretary who distributes sealed opaque numbered envelopes to the nurse manager in the operation theatre. A nurse will open the envelope only when a peroperative diagnostic evaluation has documented a type II SLAP lesion.

### Interventions

The patient will be positioned in the lateral decubitus position with lateral traction and under general anaesthesia. A standard posterior portal will be created and a diagnostic evaluation will be performed. Prior to entering the glenohumeral joint the subacromial space will be inspected and evaluated. The subacromial and the glenohumeral evaluations will be documented in a video created for each patient. An anterior working portal will be established in the rotator interval with a spinal needle for accurate placement. This portal will be used to probe the superior labrum for documentation of a type-II SLAP lesion. Arthroscopic diagnostic evaluations and treatments will be performed by a single experienced shoulder surgeon.

Following confirmation of a type II SLAP lesion, the patient will be included in the randomisation procedure. All patients will receive 20 to 40 ml of a 0.5% local anaesthetic (Marcaine) at the end of the procedure, partly to serve as a suprascapular nerve block and partly to serve as an intraarticular injection. A collar and cuff sling will be placed before the patient leaves the operating room.

#### Placebo (diagnostic arthroscopy)

Patients randomised to diagnostic arthroscopy and postoperative rehabilitation will comprise the placebo group.

#### Labral repair

Debridement of the superior glenoid rim will be performed with a motorized shaver from the anterior portal. The bioabsorbable suture anchor will be placed percutaneously, guided by a spinal needle through the myotendinous junction of the supraspinatus. From the percutaneous portal two suture anchors will be placed in the glenoid posterior to the insertion of the biceps tendon. Sutures will then be made with the use of a shuttling device from the anterior portal. Fixation will be secured with a sliding knot and three half-hitches in alternating directions. Eventually, an anterior anchor will be placed through the anterior portal. No other procedures will be performed.

#### Biceps tenodesis

Although other arthroscopic methods are described, we routinely use a mini-open technique for biceps tenodesis (14). For exact positioning of the biceps tendon, a spinal needle will be placed under arthroscopic vision, as far laterally and central as possible in the biceps tendon. A tenotomy will be performed at the biceps labrum junction. The rest of the procedure will be performed mini-open with a 2 cm skin incision with the spinal needle in the centre. In order to identify and open the biceps pulley the deltoid will be split along the muscle fibers. The biceps tendon will be identified and lifted outside of the bicipital groove. The groove will be debrided, and a metal double suture anchor with needles will be placed in the groove. One of the limbs of each suture will be placed as a simple stitch to secure sliding of the knot, and the second limb will be passed two times to secure the fixation. Approximately 2 cm of the tendon will be excised and the pulley and skin will be closed. No other procedures will be performed.

#### Post-operative rehabilitation

Patients in all three groups will have standardised, but individually adjusted rehabilitation. Elbow, wrist, and finger mobilisation and gentle pendulum exercises will be conducted, starting on the first postoperative day. A sling will be used for three weeks. Local physiotherapists or manual therapists, who are given a written detailed description of the methods and progression, will provide treatment to patients when they are discharged from the hospital. Passive techniques like massage and stretching along with core stability exercises and general physical training will be used during the first three weeks. Exercises to normalise the gleno-humeral rhythm and improve coordination and mobility will be given using sling exercise therapy [17]. Exercises to improve functional stability and muscle strength of the rotator cuff and scapular stabilising muscles will be progressively emphasised after six weeks. Sports- or job-specific rehabilitation will be given on an individual basis, usually starting three months postoperatively. Rehabilitation will continue for three to six months and will include 12-16 sessions with a therapist and about 20 sessions of self-administered exercises.

### Outcome assessment

Baseline data will include gender, age, smoking, previous treatment, duration of symptoms, MR arthrography and conventional x-rays including outlet view, and primary and secondary outcome measures.

The same blinded observer will assess all participants after the procedure at three, six, 12 and 24 months. Pain, health related quality of life, complications, and a return to sports and work will be assessed at each time point. Blinding will be evaluated by asking the patients about which treatment they perceive to have received.

Pain during activity and pain at rest (over the last week) will be measured on a 0-100 visual analogue scale (VAS), comprising a horizontal line labelled no pain at one end and worst imaginable pain at the other end.

A range of standardised, generic and specific self-report health-related quality of life measures and the clinical Rowe Score will be used. To our knowledge outcome measures have not been particularly evaluated for patients with SLAP lesions. The primary outcome measures in the present trial will be the 1988 version of the Rowe Score [18] and the Western Ontario Instability Index (WOSI)[19]. The latter has been professionally translated to Norwegian.

The Rowe Score was first described in 1978 for use in patients after they were administered the Bankart procedure for anterior shoulder dislocation [20]. Four different versions exist. We will use the 1988 version. The observer will question the patient about function and pain, and assess their stability, muscle strength, and range of motion. The Rowe Score can be weighted using either pain or stability as the main problem. Because pain is the main complaint in patients with type II SLAP lesions, we will weight pain as 25 points. Pain has five levels ranging from severe (0 points) to none (25). Stability has five levels ranging from recurrent dislocation (0) to normal shoulder stability, which includes a negative apprehension test (15). Function has five response alternatives from total disability (0) to normal function with no limitation in daily living, sports, or work (25). Range of motion is evaluated for abduction/forward flexion, internal rotation and external rotation, and it is categorised from a full range of motion (25) to less than 30° of motion (0). Muscle strength will be measured by a spring gauge, and results will be compared to the opposite shoulder and categorised from normal (10) to poor (0). The best achievable score is 100. Results are commonly classified into four categories: poor (39 points or less), fair (40 to 69 points), good (70 to 89 points), and excellent (90 to 100 points).

The WOSI is a disease-specific health related quality of life instrument developed and validated for use in patients with shoulder instability. It comprises 21 items representing four domains. The first domain covers physical symptoms and contains 10 items. The remaining domains are sports, recreation, and work (four items), lifestyle (four items), and emotions (three items). Each question is scored from 0 (best possible) to 100 on a visual analogue scale. The worst score possible is 2100. This signifies that the patient has an extreme decrease in shoulder-related health-related quality of life.

The Shoulder Instability Questionnaire (SIQ) is a disease-specific health related quality of life instrument validated for use in patients with shoulder instability [21]. It includes 12 questions (1-5 points each) with possible scores from 12 (best function) to 60 (worst function).

The EuroQuol (EQ-5 D and EQ-VAS) is a standard generic health-related quality of life instrument [22]. The EQ-5 D measures five domains (Mobility, Self-Care, Usual Activities, Pain/Discomfort, and Anxiety/Depression); each has three levels, ranging in severity from no problem, to some problem, or an extreme problem. Responses are transformed to an index and then classified into 243 (3^5^) health states, with the best imaginable state (1.0) representing the highest level of functionality.

Sickness absence data will be collected from the National Social Security Institution.

### Sample size

The main end-points are six and 24 months [12]. From clinical experience we estimated that the smallest clinically important detectable difference is 10 points on the 100 points Rowe Score. Assuming that the largest difference between treatments will be 10 units, we simulated multiple scenarios and estimated the standard deviation between means to be 14.6 units. To detect this difference between treatment groups (SD = 15, α = 0.05, β = 0.80, One-Way ANOVA) our study will require 36 patients in each group. Assuming some patients drop-out, we plan to include 40 patients in each group.

### Planned statistical analysis

Treatment groups will be examined for comparability at baseline with respect to demographic and prognostic factors. All eligible patients, regardless of their compliance with protocol (analysis by intention-to-treat) will be included in the main analyses. To asses the effect of the interventions on the endpoints (six and 24 months), analysis of covariance (ANCOVA) will be performed using the baseline values as one of the covariates. Standard regression assumptions will be assessed using diagnostic plots, Jacknife residuals, Cook's distances, and Variance inflation Factor (VIF). We will adjust for an eventual imbalance at the baseline. Corresponding post-hoc tests (Tukey's test) will be performed. To evaluate the time-course at three, six, 12, and 24 months, repeated measures will be analyzed using linear mixed models. If the number of missing values exceeds 10% in one of the groups, multiple imputations will be used to estimate the missing values. To assess the robustness of our findings the analysis will be performed with and without the imputed values.

## Discussion and conclusion

Surgery for type II SLAP lesions are performed worldwide, but published reports suggest that outcome is difficult to predict. Interventions that effectively reduce pain, improve function, and allow patients to return to sports and work are lacking. Promising results are published for both biceps tenodesis and labral repair [5,23], but the lack of a randomised design, standardised inclusion and exclusion criteria, and small study sizes, may bias these conclusions.

Few clinical trials in orthopaedic surgery include sham or placebo treatments. Two trials compared vertebroplasty [24,25] with placebo in patients with osteoporotic vertebral compression fractures, and one trial compared arthroscopic lavage, debridement, and placebo in patients with osteoarthritis of the knee [26]. Neither of these trials found that the surgical procedure was effective compared with the placebo. These trials emphasise the importance of including a placebo intervention in a randomised trial in order to improve present knowledge about mechanisms for pain reduction after surgical procedures.

## Competing interests

The authors declare that they have no competing interests.

## Authors' contributions

ØS participated in the design of the study, drafted the manuscript, and evaluated patients for inclusion and follow-up exams. CPS participated in the design of the study, operated on all patients included in the study, and helped to draft the manuscript. OR participated in the design of the study and contributed to monitoring the trial. PM participated in the design of the study, and planned and performed the statistical analyses. JIB participated in the design of the study, drafted the manuscript, and monitored the trial. All authors read and approved the final manuscript.

## Pre-publication history

The pre-publication history for this paper can be accessed here:

http://www.biomedcentral.com/1471-2474/11/228/prepub
